# Posterior Communicating Artery Configuration and Laterality of Thalamic and Lenticulostriate Infarction

**DOI:** 10.3390/neurolint18030061

**Published:** 2026-03-22

**Authors:** Junpei Nagasawa, Masamichi Hozumi, Tatsuhiro Yokoyama, Makiko Ogawa, Junya Ebina, Mari Shibukawa, Takehisa Hirayama, Osamu Kano

**Affiliations:** Department of Neurology, Toho University Faculty of Medicine, Tokyo 143-8541, Japan; n.junpei62@gmail.com (J.N.);

**Keywords:** posterior communicating artery, perforator infarction, lacunar stroke

## Abstract

**Background:** Anatomical variations in the posterior communicating artery (PCoA) are common, but their association with ischemic stroke remains unclear. In this study, we investigated the relationship between PCoA configuration and the localization of perforator infarction. **Methods:** We conducted a single-center, retrospective observational study of consecutive patients admitted with acute ischemic stroke between April 2016 and July 2023. Patients with a single, unilateral lacunar infarction confined to the thalamic or lenticulostriate artery (LSA) territory were included. PCoA configuration was assessed using time-of-flight magnetic resonance angiography and dichotomized as present (normal PCoA or fetal-type posterior cerebral artery) or absent (hypoplastic or aplastic PCoA). Using a within-patient, hemisphere-based approach, the presence of PCoA on the infarcted side was directly compared with that on the contralateral side. McNemar’s test with continuity correction was used for laterality analysis. **Results:** A total of 64 patients met the inclusion criteria, including 45 with LSA infarction and 19 with thalamic infarction. The prevalence of PCoA presence on the infarcted hemisphere was 20.0% in the LSA group and 26.3% in the thalamic group, identical to that observed on the contralateral hemisphere in each group. Within-patient comparisons revealed no significant difference in PCoA presence between infarcted and non-infarcted hemispheres in either territory (all *p* > 0.05). **Conclusions:** In patients with unilateral perforator infarction involving the thalamic or LSA territories, PCoA configuration was not associated with infarct laterality. These findings suggest that variations in PCoA anatomy have a limited influence on hemispheric vulnerability to perforator infarction, supporting the predominant role of local small-vessel pathology rather than proximal collateral anatomy in the development of lacunar stroke.

## 1. Introduction

The circle of Willis plays a critical role in maintaining cerebral perfusion through collateral circulation, particularly in the presence of arterial stenosis or occlusion [1]. Among its components, the posterior communicating artery (PCoA) represents a key anatomical connection between the anterior and posterior circulations. Anatomical variations in the PCoA are common in the general population and include hypoplasia, aplasia, and enlargement as part of a fetal-type posterior cerebral artery (PCA) configuration [2]. These variations may influence regional cerebral blood flow and collateral capacity, potentially modifying vulnerability to ischemic injury in specific vascular territories.

Previous studies have investigated the association between PCoA anatomy and ischemic stroke, yielding inconsistent and sometimes conflicting results. Although some reports have suggested that hypoplastic or absent PCoA may be associated with posterior circulation infarction, including thalamic involvement [3,4], most studies assessed stroke occurrence at the patient level. Therefore, whether PCoA configuration influences the hemispheric laterality of infarction within individual patients remains uncertain.

Thalamic and lenticulostriate artery (LSA) infarctions are typical forms of perforator infarction and are generally attributed to small vessel pathology or branch atheromatous disease, with local vascular factors playing a dominant role [5,6,7]. Unlike cortical or border-zone infarctions, they are considered less influenced by global hemodynamic compromise or proximal collateral pathways [8]. However, because the thalamus is supplied by multiple perforating arteries arising from the proximal PCA, subtle differences in collateral anatomy may theoretically influence regional perfusion reserve [9]. Similarly, whether proximal arterial configurations affect the hemispheric vulnerability of LSA infarction remains unclear.

Importantly, the potential contribution of PCoA anatomy to the laterality of perforator infarction has not been adequately examined. Most prior studies relied on inter-individual comparisons, which are susceptible to confounding. In contrast, a within-patient hemispheric analysis allows direct assessment of whether infarction preferentially occurs on the side with a specific arterial configuration.

Therefore, the present study aimed to investigate the relationship between PCoA configuration and the laterality of unilateral thalamic and lenticulostriate infarction using a patient-level, side-by-side comparison approach.

## 2. Method

### 2.1. Study Design and Patient Selection

This was a single-center, retrospective observational study conducted at a tertiary care hospital. We reviewed consecutive patients admitted with acute ischemic stroke between April 2016 and July 2023. By including all consecutively admitted patients within the predefined study period, we aimed to minimize selection bias and reflect real-world clinical practice.

Eligible patients were 18 years of age or older and had a diagnosis of acute ischemic stroke confirmed by brain magnetic resonance imaging (MRI). Stroke subtypes were classified according to the Trial of ORG 10,172 in Acute Stroke Treatment (TOAST) criteria to ensure standardized etiological categorization.

From the overall stroke cohort, patients were selected if they had a single, unilateral lacunar infarction located in either the thalamic territory or the lenticulostriate artery (LSA) territory, as identified on diffusion-weighted imaging (DWI). Lacunar infarction was defined as a small, deep infarction consistent with perforator artery involvement, without cortical extension, in accordance with established radiological criteria for small-vessel disease. The restriction to solitary and unilateral lesions was essential for the laterality-based analytical design, which required clear hemispheric distinction between infarcted and non-infarcted sides.

Patients were excluded if they had bilateral infarctions, multiple acute infarcts, infarctions involving both thalamic and LSA territories, or non-lacunar infarctions. These exclusions were applied to avoid ambiguity in assigning hemispheric laterality and to reduce heterogeneity in stroke mechanisms. Additional exclusion criteria included large territorial infarctions, significant (>50%) stenosis or occlusion of the internal carotid artery, vertebral artery, basilar artery, or posterior cerebral artery (PCA), and cardioembolic stroke, including cases with atrial fibrillation, severe heart failure, or other high-risk cardiac sources of embolism. These criteria were applied to minimize the influence of large-artery hemodynamic compromise or embolic mechanisms, which could confound the assessment of perforator infarction laterality.

Furthermore, patients with arterial dissection, vasculitis, antiphospholipid antibody syndrome, coagulation disorders, or other identified specific causes of ischemic stroke were excluded to ensure a relatively homogeneous cohort of presumed small-vessel or branch atheromatous disease. Patients with insufficient imaging quality that precluded reliable evaluation of the PCoA were also excluded. Demographic information and vascular risk factors, including hypertension, diabetes mellitus, dyslipidemia, and smoking status, were retrospectively obtained from electronic medical records using standardized definitions.

### 2.2. Imaging Analysis and Infarction Classification

All patients underwent brain MRI using Siemens scanners (1.5-T or 3-T systems; Siemens Healthineers, Erlangen, Germany). MRI was performed within 7 days of stroke onset and included DWI, apparent diffusion coefficient (ADC) maps, fluid-attenuated inversion recovery (FLAIR), and time-of-flight magnetic resonance angiography (TOF-MRA). TOF-MRA is a flow-dependent, non-contrast technique that visualizes intracranial arteries based on inflowing unsaturated spins. Although widely used in clinical practice, TOF MRA may underestimate vessel caliber in conditions of low flow or slow velocity and is limited by spatial resolution compared with digital subtraction angiography. The use of both 1.5-T and 3-T scanners reflects routine clinical practice; higher field strength may provide an improved signal-to-noise ratio and spatial resolution, potentially enhancing visualization of small-caliber vessels such as the PCoA.

All MRI examinations were independently reviewed by an experienced stroke neurologist who was blinded to clinical information and infarct laterality at the time of image assessment. Image review was performed using standardized radiological criteria to ensure consistent lesion classification. In cases of uncertainty regarding lesion location or vascular territory, images were re-evaluated jointly to achieve consensus.

Acute infarction was defined as a hyperintense lesion on diffusion-weighted imaging (DWI) with corresponding hypointensity on the apparent diffusion coefficient (ADC) map, consistent with restricted diffusion. FLAIR images were also examined to confirm lesion conspicuity and exclude chronic ischemic changes or leukoaraiosis that could mimic acute pathology.

**Figure 1:**
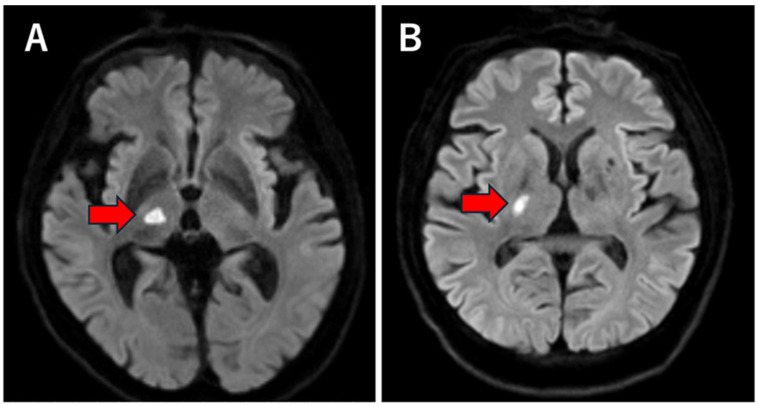
Representative axial diffusion-weighted imaging (DWI) of perforator infarction. (**A**) Axial DWI demonstrating a small, unilateral acute infarction in the LSA territory involving the internal capsule/basal ganglia (arrow). (**B**) Axial DWI demonstrating a unilateral acute thalamic infarction confined to the thalamus without cortical involvement (arrow). DWI, diffusion-weighted imaging; LSA, lenticulostriate artery.

Thalamic infarction was defined as an acute ischemic lesion confined strictly to the thalamus without extension into adjacent midbrain or cortical structures (Figure 1A). LSA infarction was defined as an infarction located in the basal ganglia, internal capsule, or corona radiata, consistent with the vascular territory of the lenticulostriate arteries arising from the M1 segment of the middle cerebral artery (Figure 1B). These territories were identified based on established anatomical and radiological descriptions of perforator artery distribution.

To ensure validity of hemispheric laterality analysis, only patients with strictly unilateral and solitary lesions identified on acute-phase MRI were included. Cases with equivocal bilateral signal changes, multifocal lesions, or lesions crossing vascular territory boundaries were excluded to avoid misclassification and preserve a clear within-patient hemispheric comparison framework.

### 2.3. Assessment of PCoA Configuration

Anatomical variations in the posterior portion of the circle of Willis were evaluated using time-of-flight MRA. First, the configuration of the PCA was assessed. Fetal-type PCA was defined as a PCA predominantly supplied by the internal carotid artery, characterized by a P1 segment that was absent or smaller in caliber than the ipsilateral PCoA.

**Figure 2:**
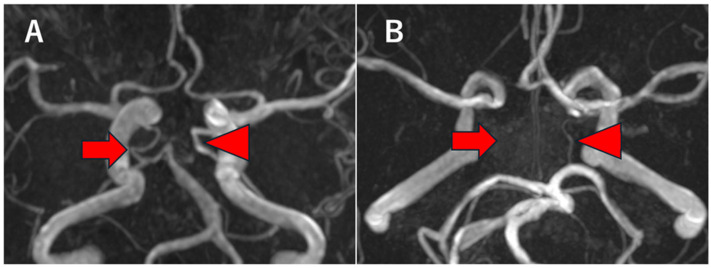
Representative axial time-of-flight magnetic resonance angiography (TOF-MRA) images demonstrating different configurations of the posterior communicating artery (PCoA). (**A**) Normal and fetal-type PCoA. The arrow indicates a normal PCoA, whereas the arrowhead indicates a fetal-type posterior cerebral artery supplied predominantly by the internal carotid artery. (**B**) Absence and hypoplasia of the PCoA. The arrow indicates an aplastic PCoA, while the arrowhead indicates a hypoplastic PCoA.

Subsequently, the morphology of the PCoA itself was evaluated. Normal PCoA was defined as a continuously visualized artery with a diameter of ≥1.0 mm on time-of-flight MR angiography (Figure 2A). This threshold was adopted based on prior MRA studies, such as Haghighimorad et al., who defined PCoA hypoplasia/aplasia as a diameter <1 mm or non-visualization on TOF MRA [3]. Although diameter cut-offs are not universally standardized across studies, the <1.0 mm criterion has been used as a practical reference in morphological and imaging analyses of the circle of Willis. Because of the limited spatial resolution and flow-dependent nature of MR angiography, hypoplastic and aplastic PCoA could not be reliably distinguished; therefore, these conditions were analyzed together and defined as a PCoA diameter <1.0 mm or non-visualization of the PCoA (Figure 2B). For the purpose of laterality analysis, PCoA configuration was dichotomized as PCoA present (including normal PCoA and fetal-type PCA) or PCoA absent (including hypoplastic or aplastic PCoA), reflecting the presence or absence of a functionally patent communicating artery.

The dichotomized classification was adopted to reflect the functional presence or absence of a communicating pathway rather than subtle differences in vessel caliber. Given that the physiological role of the PCoA as a collateral channel depends primarily on whether a patent conduit exists, a binary classification was considered appropriate for laterality analysis. While vessel diameter represents a continuous variable, minor variations above the 1.0 mm threshold are unlikely to substantially alter hemispheric collateral capacity in the absence of proximal large-artery disease.

Diameter measurements were performed manually using the electronic caliper tool on axial source images of TOF MRA at the level where the PCoA was most clearly visualized. Measurements were conducted by an experienced neurologist blinded to the infarct laterality. Formal interobserver reproducibility analysis was not performed.

Laterality was defined as the hemispheric relationship between infarction and ipsilateral PCoA configuration. In patients with unilateral thalamic or LSA infarction, the presence or absence of PCoA on the infarcted side was directly compared with that on the contralateral, non-infarcted side, allowing a within-patient, hemisphere-based analysis.

### 2.4. Statistical Analysis

We analyzed the association between PCoA configuration and infarct laterality in patients with unilateral thalamic or LSA infarction. PCoA configuration was dichotomized as PCoA present (normal PCoA or fetal-type PCA) or PCoA absent (hypoplastic or aplastic PCoA), and the frequency of PCoA presence/absence was evaluated for the infarcted and contralateral hemispheres.

Categorical variables are presented as numbers (percentages), and continuous variables are presented as mean ± standard deviation or median with interquartile range, as appropriate. Baseline characteristics were compared between the thalamic and LSA infarction groups using the χ^2^ test or Fisher’s exact test for categorical variables and the unpaired *t*-test or Mann–Whitney U test for continuous variables, as appropriate.

For the primary laterality analysis, within-patient comparisons between the infarcted and contralateral hemispheres were performed using McNemar’s test. This test was selected because the primary objective of the study was to evaluate whether PCoA configuration differed between the infarcted and contralateral hemispheres within the same individual. In this paired design, each patient contributed two dependent observations (infarcted vs. contralateral hemisphere), and therefore standard chi-square tests for independent samples would not have been appropriate. McNemar’s test specifically evaluates discordant pairs in paired categorical data and directly assesses whether the probability of PCoA presence differs between hemispheres within subjects. By focusing on within-patient comparisons, this approach minimizes inter-individual confounding related to age, vascular risk factors, and overall cerebrovascular anatomy, as each patient serves as his or her own control. Because McNemar’s test is driven by the number of discordant pairs, the effective sample size for this analysis corresponds to the number of patients with hemispheric mismatch in PCoA configuration. Continuity correction was applied to provide a more conservative estimate in the context of relatively small discordant counts.

All statistical tests were 2-sided, and *p*-values <0.05 were considered statistically significant. Statistical analyses were performed using JMP Student Edition, version 18 (SAS Institute Inc., Cary, NC, USA). McNemar’s exact test was calculated using GraphPad QuickCalcs (GraphPad Software, San Diego, CA, USA; accessed on 21 March 2026).

## 3. Results

### 3.1. Patient Selection and Baseline Characteristics

During the study period, 666 consecutive patients were admitted with acute ischemic stroke and underwent brain MRI. From this overall stroke cohort, patients with single, unilateral lacunar infarction confined strictly to either the LSA territory or the thalamic territory were identified according to the predefined imaging criteria. After applying the inclusion and exclusion criteria, a total of 64 patients met the final eligibility criteria, representing 9.6% of the overall ischemic stroke cohort. Among these, 45 patients had LSA infarction and 19 patients had thalamic infarction.

The relatively small proportion of eligible patients reflects the stringent selection criteria, which were designed to ensure a homogeneous cohort suitable for within-patient hemispheric comparison. In particular, patients with multiple acute infarcts, bilateral lesions, or significant large-artery stenosis were excluded to minimize confounding mechanisms and preserve a clear laterality-based analytical framework.

Baseline clinical characteristics of the two groups are summarized in Table 1. The prevalence of diabetes mellitus was significantly higher in patients with thalamic infarction than in those with LSA infarction (10 [52%] vs. 10 [22%], *p* = 0.03). Similarly, current smoking was more frequent in the thalamic infarction group compared with the LSA infarction group (11 [57%] vs. 12 [26%], *p* = 0.03). These differences suggest potential heterogeneity in vascular risk profiles between the two perforator territories. Other baseline characteristics, including age, sex distribution, hypertension, and dyslipidemia, were not significantly different between the two groups, indicating broadly comparable demographic and clinical backgrounds aside from the above-mentioned factors.

### 3.2. PCoA Configuration and Infarct Laterality

The prevalence of PCoA presence on the infarcted hemisphere was 9 of 45 patients (20.0%) in the LSA infarction group and 5 of 19 patients (26.3%) in the thalamic infarction group.

Similarly, PCoA presence on the contralateral hemisphere was observed in 9 patients (20.0%) with LSA infarction and 5 patients (26.3%) with thalamic infarction.

In the LSA infarction group, bilateral PCoA presence was observed in 5 patients (11%), whereas bilateral absence was observed in 32 patients (71%). The remaining patients demonstrated unilateral PCoA presence.

In the thalamic infarction group, bilateral PCoA presence was observed in 4 patients (21%), whereas bilateral absence was observed in 13 patients (68%). The remaining patients showed unilateral PCoA presence.

Within-patient comparisons confirmed the absence of hemispheric imbalance. In the LSA group, the number of discordant pairs were equal (4 vs. 4), and McNemar’s test with continuity correction showed no significant difference (χ^2^ = 0.125, *p* = 0.724). Likewise, in the thalamic group, discordant pairs were also balanced (1 vs. 1), with no significant difference detected (χ^2^ = 0.50, *p* = 0.48).

Thus, PCoA configuration was not associated with infarct laterality in patients with unilateral perforator infarction (Table 2).

## 4. Discussion

In this retrospective study of patients with unilateral lacunar infarction involving the LSA or thalamic regions, we found that the presence of the PCoA did not differ between the infarcted and contralateral hemispheres. These findings suggest that PCoA anatomy may not be a major determinant of hemispheric vulnerability to perforator infarction in either vascular region. Rather, our results support the concept that local small-vessel pathology plays a predominant role in the development of isolated perforator infarction.

Anatomical variations in the PCoA are common in the general population and constitute an important component of variability within the posterior portion of the circle of Willis. The circle of Willis functions as a potential collateral network that can redistribute cerebral blood flow in the setting of proximal arterial stenosis or occlusion. Within this network, the PCoA represents the principal anastomotic channel connecting the anterior and posterior circulations. Therefore, its configuration has long been considered relevant to cerebral hemodynamics and ischemic tolerance.

Variations in the PCoA include absence (aplasia), hypoplasia, normal adult-type configuration, and fetal-type PCA, in which the PCA is supplied predominantly by the internal carotid artery via a prominent communicating artery. Embryologically, the fetal-type PCA reflects persistence of the primitive carotid–PCA connection, and its presence alters the balance of flow between the anterior and posterior circulations. Previous anatomical and imaging studies have reported that fetal-type PCA variants occur in approximately 20–30% of individuals in general populations [10,11], highlighting that this configuration is not rare but represents a frequent anatomical variant.

In contrast, the reported prevalence of unilateral PCoA hypoplasia or aplasia ranges widely from 8% to 28.7%, while that of bilateral PCoA hypoplasia or aplasia ranges from 3.7% to as high as 47.5% across different studies [2]. This substantial variability likely reflects differences in study populations, imaging modalities, spatial resolution, and diagnostic criteria used to define PCoA morphology. Moreover, because PCoA visualization on noninvasive imaging is flow-dependent, reported prevalence may be influenced by physiological as well as anatomical factors.

Consequently, although PCoA configuration represents a frequent anatomical variation with potential hemodynamic implications, its clinical significance—particularly with respect to ischemic vulnerability in specific vascular territories—remains incompletely understood. While its role in large-artery occlusive disease has been extensively discussed, far less is known about whether PCoA anatomy influences the laterality or susceptibility of small, perforator-type infarction.

Several previous studies have focused on the hemodynamic implications of PCoA configuration in ischemic stroke. Because the circle of Willis functions as a dynamic collateral network rather than a static anatomical structure, variations in its configuration may substantially influence cerebral perfusion under pathological conditions.

In the presence of a fetal-type PCA, the internal carotid artery (ICA) supplies not only the anterior circulation but also a substantial portion of the PCA territory via a large and functionally dominant PCoA. In this configuration, the P1 segment of the PCA is either absent or markedly hypoplastic, and posterior cerebral perfusion becomes dependent primarily on the carotid system. As a result, the hemodynamic territories of the anterior and posterior circulations are partially coupled. Consequently, severe ICA stenosis or occlusion may simultaneously compromise perfusion in both anterior and posterior territories, reducing the capacity for compensatory flow redistribution from the vertebrobasilar system.

From a physiological standpoint, effective collateral compensation depends on pressure gradients across communicating arteries and the presence of alternative inflow sources. In fetal-type PCA, the absence of a well-developed P1 segment limits posterior-to-anterior collateral recruitment through the basilar artery. This may diminish perfusion reserve, particularly under conditions of systemic hypotension or proximal large-artery disease. Therefore, this anatomical configuration has been reported to be associated with reduced ischemic tolerance and larger infarct volumes in the setting of carotid occlusive disease [11]. Autopsy studies have shown that fetal-type PCA is more frequently observed in brains with infarction than in those without, suggesting a possible association between circle of Willis anatomy and ischemic stroke susceptibility [12,13]. These findings support the concept that certain configurations of the posterior circulation may predispose individuals to ischemia when major arterial inflow is compromised. However, most of these observations were derived from studies of large-artery atherosclerosis or embolic stroke, where global hemodynamic compromise and collateral recruitment play a central role in determining tissue viability.

On the other hand, several studies have focused on the absence or hypoplasia of the PCoA, reporting that an incomplete posterior collateral pathway may be associated with unfavorable hemodynamic conditions in ischemic stroke [14]. In the setting of large-artery stenosis or occlusion, the capacity of the circle of Willis to redistribute flow depends not only on the presence of communicating arteries but also on their caliber and functional patency. Hypoplasia or aplasia of the PCoA may restrict posterior-to-anterior collateral recruitment, particularly when the vertebrobasilar system is required to compensate for compromised carotid inflow. These observations have primarily been described in the context of large-artery stenosis or occlusion and cortical infarction, in which collateral circulation plays a critical role in maintaining cerebral perfusion [15]. In such scenarios, regional perfusion pressure gradients determine the direction and magnitude of collateral flow, and the absence of a communicating artery may exacerbate hypoperfusion in border-zone territories. Therefore, anatomical incompleteness of the posterior portion of the circle of Willis has been interpreted as a potential risk factor for hemodynamic instability. Importantly, however, the relevance of these mechanisms to perforator infarction is less clear. Unlike cortical territories, which may benefit from extensive leptomeningeal collateral networks, perforating arteries supplying deep structures such as the thalamus and basal ganglia are typically end-arterial branches with limited collateralization. Consequently, even if proximal collateral pathways are compromised, the extent to which this translates into altered perfusion at the level of small penetrating arteries remains uncertain.

Haghighimorad et al. reported that aplasia or hypoplasia of the ipsilateral PCoA was associated with a significantly higher incidence of thalamic territory infarction compared to patients without such variation (16.8% vs. 2.4%, *p* = 0.024) [3]. Chuang et al. reported that PCoA hypoplasia was more prevalent in patients with ischemic stroke than in controls and was frequently associated with ipsilateral thalamic lacunar infarction, even in the absence of internal carotid artery occlusion [4]. These studies have suggested that hypoplasia of the PCoA may result in insufficient local collateral flow to the thalamic perforating arteries (posterior thalamoperforators/posterior choroidal artery system), thereby predisposing the thalamus to a low-flow state and subsequent lacunar infarction.

Conversely, several studies have reported no significant association between PCoA configuration and the occurrence of ischemic stroke.

For example, a CT angiography study found no excess of fetal-origin PCA on the symptomatic side among patients with PCA-territory TIA/infarction, arguing against fetal-origin PCA as an independent risk factor for PCA-territory ischemia [16]. Likewise, in an ischemic-stroke cohort, the presence of fetal-type PCA ipsilateral to stroke was not associated with PCA-territory involvement [17]. In the context of small-vessel disease, Circle of Willis configuration was not associated with early neurological deterioration after lacunar stroke [18].

Taken together, previous studies have primarily evaluated the relationship between PCoA configuration and stroke risk, infarct size, or infarct distribution across patient populations.

In contrast, the present study focused on within-patient hemispheric comparisons to directly assess whether PCoA configuration influences the laterality of perforator infarction. This approach minimizes inter-individual confounding related to vascular risk factors and anatomical variability.

Using this laterality-based approach, we found that the presence or absence of the PCoA did not differ between infarcted and contralateral hemispheres in either LSA or thalamic infarction.

When analyzed separately, no significant hemispheric lateralization according to PCoA configuration was observed in the LSA infarct group. Similarly, no significant lateralization was identified in the thalamic infarct group; however, the sample size was limited in this subgroup, and subtle associations cannot be excluded. Given the distinct vascular anatomy supplying the thalamus (including P1/P2 perforators and potential Percheron-like variants) compared with the predominantly M1-derived lenticulostriate arteries, these results should be interpreted within each vascular territory.

These findings suggest that, unlike large-artery occlusive disease, the development of perforator infarction may be driven predominantly by local small-vessel pathology rather than by proximal collateral anatomy at the level of the circle of Willis.

Taken together, the absence of hemispheric differences in both subgroups suggests that PCoA configuration has limited impact on the laterality of perforator infarction overall, although statistical power was limited particularly in the thalamic subgroup.

Several limitations should be acknowledged. First, this was a single-center retrospective study with a relatively small sample size, particularly in the thalamic infarction group, which may limit statistical power. Because this was a retrospective study based on consecutively admitted patients, selection bias was minimized by including all eligible cases within the predefined study period. The single-center design ensured consistency in imaging protocols, diagnostic evaluation, and stroke management pathways throughout the study period. However, this design may limit generalizability to other institutions with different imaging protocols or patient demographics.

Second, PCoA configuration was assessed using time-of-flight MR angiography, which may underestimate small-caliber vessels due to limited spatial resolution. Third, detailed quantitative assessment of P1 and PCoA diameters was not performed, and functional collateral flow could not be directly evaluated. The dichotomization of PCoA morphology into “present” versus “absent” may have introduced misclassification bias, as vessel caliber represents a continuous variable. However, given the limited spatial resolution and flow-dependent nature of TOF MRA, a simplified binary classification was considered a pragmatic and reproducible approach.

In addition, potential asymmetry of subclinical atherosclerosis or microangiopathy between hemispheres was not assessed. Even in the absence of overt large-vessel stenosis, subtle side-to-side differences in small-vessel pathology may influence the vulnerability of perforating arteries and could theoretically mask a weak association between PCoA configuration and infarct lateralization. Finally, multivariable analyses were not conducted, and residual confounding cannot be excluded. Although a combined conditional logistic regression analysis including vascular territory was considered, the limited sample size—particularly in the thalamic subgroup—precluded stable modeling and raised concerns about potential overfitting. Therefore, we retained the predefined paired analyses within each subgroup.

Furthermore, because of the restrictive inclusion criteria, the sample size was relatively small, particularly in the thalamic infarction group, limiting statistical power to detect subtle associations. Because McNemar’s test depends on the number of discordant pairs, power was especially limited in the thalamic subgroup. In the LSA subgroup (8 discordant pairs), statistical significance at α = 0.05 would require an extreme imbalance (8 vs. 0; 17.8% absolute difference), whereas in the thalamic subgroup (2 discordant pairs), even maximal imbalance would not reach significance. Thus, our findings argue against large lateralization effects but do not exclude subtle associations, and should be interpreted with caution.

## 5. Conclusions

In patients with unilateral lacunar infarction involving the LSA or thalamic territories, PCoA anatomy was not associated with infarct laterality in either vascular territory. Although statistical power was limited in the thalamic subgroup, the consistent absence of hemispheric differences suggests that variations in PCoA configuration may have limited influence on perforator infarct lateralization. These findings support the predominant role of local small-vessel pathology and vascular risk factors in the development of lacunar stroke.

## Figures and Tables

**Table 1:** Baseline characteristics of patients with unilateral lacunar infarction involving the LSA or thalamic territories.

	LSA Infarction (*n* = 45)	Thalamic Infarction (*n* = 19)	*p*-Value
Male, *n* (%)	28 (62)	13 (68)	0.85
Age (mean ± SD) years	66.2 ± 13.3	70.7 ± 13.4	0.16
NIHSS (mean ± SD) points	1.64 ± 2.0	1.68 ± 1.1	0.90
Hypertension, *n* (%)	31 (68)	15 (78)	0.60
Dyslipidemia, *n* (%)	20 (44)	8 (42)	0.91
Diabetes, *n* (%)	10 (22)	10 (52)	0.03
CKD, *n* (%)	5 (11)	1 (5)	0.79
Alcohol consumption, *n* (%)	13 (28)	6 (31)	0.93
Smoker, *n* (%)	12 (26)	11 (57)	0.03
SAS, *n* (%)	1 (2)	3 (15)	0.13

LSA, lenticulostriate artery; NIHSS, National Institutes of Health Stroke Scale; CKD, chronic kidney disease; SAS, sleep apnea syndrome. Data are presented as mean ± standard deviation or number (%). Categorical variables were compared using the chi-square test or Fisher’s exact test, as appropriate. Continuous variables were compared using Student’s *t*-test or the Mann–Whitney U test, as appropriate.

**Table 2:** PCoA presence on infarcted and contralateral hemispheres according to infarction territory.

	Infarction Side	Contralateral Side	*p* Value
LSA infarction (*n* = 45), *n* (%)	9 (20)	9 (20)	>0.05
Thalamic infarction (*n* = 19), *n* (%)	5 (26)	5 (26)	>0.05

PCoA, posterior communicating artery; LSA, lenticulostriate artery.

## Data Availability

The original contributions presented in this study are included in the article.
